# IL28B Gene Polymorphisms and US Liver Fatty Changes in Patients Who Spontaneously Cleared Hepatitis C Virus Infection

**DOI:** 10.1371/journal.pone.0067301

**Published:** 2013-08-01

**Authors:** Gloria Taliani, Martina Spaziante, Elisa Biliotti, Marina Borro, Donatella Palazzo, Stefania Grieco, Cristiana Franchi, Giancarlo Iaiani, Caterina Furlan, Valentina Gallinaro, Maurizio Simmaco

**Affiliations:** 1 Clinica Malattie Infettive e Tropicali, Department of Clinical Medicine, Policlinico Umberto I, Sapienza University of Rome, Rome, Italy; 2 Advanced Molecular Diagnostics Unit, Sant'Andrea Hospital, Dipartimento Neuroscienze Salute Mentale Organi di Senso, Sapienza University of Rome, Rome, Italy; Saint Louis University, United States of America

## Abstract

**Background:**

Recent clinical studies have shown that the presence of CC genotype in the rs12979860 region of IL28B gene is associated with an increase in the probability of spontaneous clearance of hepatitis C virus (HCV). Moreover, IL28B polymorphism seems to influence the probability of developing liver steatosis in chronic HCV patients.

**Aims:**

The aims of our clinical study were 1) to verify the distribution of IL28B genotypes (CC, CT or TT) among subjects with spontaneous clearance of HCV infection and 2) to examine the correlation between IL28B polymorphism and hepatic steatosis among these subjects.

**Methods and patients:**

We enrolled 41 subjects with spontaneous resolution of HCV infection (detectable serum anti-HCV but undetectable HCV-RNA) and 134 healthy controls from the same geographical area. The IL28B single-nucleotide polymorphism (SNP) rs12979860 was genotyped by using a Pyrosequencing™ technique. The presence of steatosis was assessed by liver biopsy or ultrasound examination in the 41 study subjects.

**Results:**

CC, CT and TT-genotypes of the SNP rs1979860 were found in 66%, 24% and 10% of the subjects who spontaneously cleared HCV and in 31%, 54% and 15% of controls, respectively (p = 0.0003). Among the study subjects, females with CC-genotype were significantly more represented (p = 0.02). Hepatic steatosis did not correlate with IL28B genotype (p = 0,14) but only with a high body mass index (BMI) value (p = 0.03).

**Conclusions:**

Female subjects carrying IL28B CC-genotype are significantly more represented among Italian patients who spontaneously cleared HCV infection. In addition, among these subjects, the presence of liver steatosis does not correlate with IL28B genotype but is solely related to the occurrence of high BMI. Thus, the association between IL28B polymorphism and steatosis in chronic HCV patients requires the presence of active HCV replication to occur, while in subjects who have cleared the infection, the mechanism(s) inducing liver steatosis are independent from IL28B profile.

## Introduction

Hepatitis C virus (HCV) is a positive-stranded RNA virus and one of the most common blood borne infectious agent worldwide. HCV infection induces a wide range of innate and adaptive immune responses [1]–[2] but eventually the majority of infected individuals develop chronic HCV infection, while only approximately 30–40% of them spontaneously clear the virus [3]. In these subjects, serum anti-HCV antibodies remain detectable for a long time without evidence of circulating HCV-RNA.

Several clinical studies demonstrated that viral [4]–[5], environmental [6] and host [6], [7] factors have a significant role in protecting against the development of chronic HCV infection. However, the mechanisms that address host's response to the spontaneous clearance of the virus are still not perfectly known.

Recent genome-wide association studies (GWAS) have shown a single-nucleotide polymorphisms (SNPs) around the gene coding for IFN-λ-3 (or IL28B), located on chromosome 19q13, to be associated with spontaneous virus clearance in patients with HCV infection. Such polymorphism could explain at least in part the heterogeneity in HCV clearance across different ethnicity and across individuals [8].In caucasian population, rs12979860 was widely examined and was shown to be associated with a higher probability of spontaneous clearance of HCV [7], [9]. Across all examined cohorts, subjects with the CC genotype were three times more likely to clear HCV relative to non-CC, i.e. heterozygotes (CT) and homozygotes for the minor allele (TT).

In addition, a significant association has been found between IL28B CT or TT genotypes and hepatic steatosis in chronic HCV infection [19–11], which is probably mediated by a higher expression of IFN-stimulated genes (ISGs) in carriers of such genotypes [12].

Three SNPs have been identified and studied worldwide: rs12979860, rs12980275 and rs8099917, which are in strong linkage disequilibrium (LD) with each other [13]. We have chosen to investigate rs12979860 polymorphism because the association of this SNP either with spontaneous clearance of HCV either with disturbances of lipid metabolism and hepatic steatosis are the most widely studied in Caucasian subjects [10], [14]–[15] and it would be easier to compare our results with others already published.

Therefore, we genotyped this SNP in a cohort of individuals who spontaneously cleared the virus and compared the distribution of the alleles with that of a HCV-negative control population from the same geographical origin. In addition, we explored the association of IL28B genotypes with steatosis in subjects with spontaneous resolution of HCV infection, to evaluate whether it is demonstrable also in the absence of actively replicating HCV.

In the present study we confirmed that subjects who spontaneously cleared HCV infection carry the CC genotype with higher frequency compared to controls. Moreover, among subjects without active replication of HCV, we could not demonstrate an association between hepatic steatosis and IL28B genotype, indicating that the lipid metabolism disturbance observed in CT/TT patients with chronic HCV infection requires the presence of actively replicating HCV virus to occur. Therefore it is not independent from, but presumably mediated by the poor control on HCV replication that patients with the unfavourable IL28B genotype exert.

## Patients and Methods

### Patients and controls

Our study was done in a cohort of 41 subjects (study subjects) enrolled from January 2008 to January 2012 at the Department of Tropical and Infectious Diseases of the Sapienza University of Rome. The established criteria for the enrolment were: repeatedly positive anti-HCV antibodies by third generation ELISA test (Abbott HCV EIA 2.0, Abbott Laboratories, Abbott Park, Illinois) in at least three examinations done 3–6 months apart; negative serum HCV-RNA by PCR (Abbott Real Time HCV quantitative assay, Abbott Laboratories, Abbott Park, Illinois, with a detection limit of 10 IU/ml) in the last three consecutive samples obtained at 3–6 months interval during the follow-up (9–60 months); and no history of antiviral treatment.

In order to assess a possible difference in prevalence of rs12979860 alleles between individuals who spontaneously cleared HCV and the general population, we compared the results of the study subjects to those obtained in healthy control subjects of the same ethnicity and from the same geographical context, to avoid any bias due to the not uniform distribution of IL28B alleles among different populations and ethnic groups [16]. The control population consisted of 134 consecutively recruited health-care workers without ALT abnormalities or any other relevant pathology, who tested negative for HCV, HBV and HIV.

All the subjects have been thoroughly informed about the aim of the study and they gave a written consent to undergo the analysis of their DNA from peripheral blood mononuclear cells (PBMCs). The study was performed in accordance with the ethical guidelines of the 1975 Declaration of Helsinki and was approved by the local ethical committee. The study was performed according to the GCP rules and approved from the Ethical Committee of the Policlinico Umberto I.

### Assessment of steatosis

Steatosis was assessed by liver biopsy in study subjects who had raised ALT levels in at least one occasion during the follow-up. In the remaining subjects, who were HCV-RNA negative and had persistently normal ALT levels, a liver biopsy was considered unethical, therefore a careful ultrasound evaluation was done to evaluate the presence of liver steatosis. To this end, the following five main criteria were employed [17]. 1) Presence of hyperechogenic parenchyma, with fine, tightly packed echoes: the so-called parenchymal brightness; 2) evident ultrasonographic contrast between hepatic parenchyma and right renal cortex; 3) deep beam attenuation with blurred, gray or indistinguishable diaphragm; 4) presence of bright walls of small intrahepatic vessels during the first minutes of the US examination; 5) poor definition of gallbladder wall (blurred or absent). The presence of at least two criteria was necessary to confirm the presence of steatosis [17].

### IL28B genotyping

Genotyping of the IL28B (rs12979860) was conducted in a blinded fashion relative to the subject being a case or a control. Total DNA, extracted from whole blood samples, was subjected to the Pyrosequencing™ technique, using PSQ96 instrument from Pyrosequencing™ AB [18]. This sequencing method is based on real-time pyrophosphate detection, which may be advantageous compared to other sequencing methodologies. First of all the sequencing primer was hybridized to an immobilized single-stranded DNA template. Then we loaded into a reagent cartridge (PSQ96. SNP Reagent Kit, Pyrosequencing AB) a mixture of four enzymes (DNA polymerase, ATP sulfurylase, luciferase, and the nucleotide-degrading enzyme apyrase), the substrate luciferin and the four separate deoxynucleotides. After the dispensation of the enzyme reagents and the substrate into the microtiter, each different nucleotide has been dispensed sequentially by ink-jet technology. For SNP detection the dispensation order was pre-defined and automatically selected for rs 12979860 SNP, using the SNP Entry module of the SNP Analysis Software (Pyrosequencing AB). The result of these cycles of nucleotide delivery was a pyrophosphate's (PPi) release at every nucleotide incorporated. Subsequently, the pyrophosphate is converted to ATP by ATP sulfurylase and light generated from the subsequent reaction, catalyzed by luciferase, was recorded by a CCD camera. Between two cycles, unincorporated nucleotides were degraded by apyrase. We used as positive controls presynthesized DNA template, corresponding to homozygotes and heterozygotes for the SNP, as well as template and primer oligonucleotides obtained from Pyrosequencing™ AB. Genotyping calls were manually inspected and verified prior to release. Hardy-Weinberg Equilibrium was assessed.

### Statistical analysis

SPSS statistical software, Version 17.1 (SPSS Inc. Chicago, Illinois, USA) was used. Chi-square testing or Fisher's exact test was applied when appropriate for testing the association of IL28B genotype with all other characteristics. Continuous variables are presented as mean ± standard deviation (SD), and Mann-Whitney non-parametric test is employed for comparison. IL28B genotypes were coded either to test each genotype (CC, CT and TT), either to test a recessive model, comparing patients homozygous (CC) to those with one or no copies of the C allele (CT/TT). Univariate and multivariate logistic regression analyses were performed to evaluate the correlation of steatosis with IL28B genotype and other variables.

## Results

### Demographic and epidemiological characteristics

Our study cohort consisted of 41 subjects (mean age 56 years ± DS 15, range 23–82 years), 95% Caucasian (39/41), the majority were female (63%) and mean BMI was 24.1 kg/m^2^±4 (range 15.6–35 kg/m^2^). The characteristics of the subjects are reported in Table 1. Thirty-five subjects had normal ALT levels (85%), while 6 had values between 1,2 and 2 times the upper normal values. All subjects were negative for HIV and for hepatitis B surface antigen (HBsAg), 10 (26%) reported previous vaccination against hepatitis B virus (HBV), and 10 (26%) had anti-HBs and anti-HBc antibodies indicating spontaneous resolution of HBV infection.

**Table 1:** Baseline Characteristics of the 41 subjects with spontaneous resolution of HCV infection enrolled in the study.

Baseline Characteristics	*Mean* ± *SD*	*N (%)*
Age (years)	56,2±15	
Female sex		24 (63)
BMI (Kg/cm^2^)	24,1±4	
BMI >25 Kg/cm^2^		18 (44)
Abnormal transaminases values		6 (16)
AST (IU/ml)	36±3	
ALT (IU/ml)	43±3	
Cholesterol Total mg/dl	198.6±45	
HDL Cholesterol mg/dl	53.6±12	
LDL Cholesterol mg/dl	125.5±39	
Tryglicerides mg/dl	124.1±55	
Hepatic Steatosis		25 (66)
Documented positive HCV viremia in the past		11 (27)
HCV genotype identified*		4 (10)
Time from the first detection of anti-HCV (years)	10.7±6	
Anti-HBc and anti-HBs positive		10 (26)
Only anti-HBs positive (vaccine recipients)		10 (26)
Risk Factors for HCV infection#		
Previous intravenous illicit drugs use		3 (7)
Blood or blood components transfusions		14 (34)
Heath Care Workers		4 (10)
Family member of HCV positive		14 (34)
Major surgical interventions		18 (44)

* : 2 genotype 1b, 1 genotype 1a, 1 genotype 4.

#: not mutually exclusive.

HCV infection was diagnosed between 2 and 25 years before the enrolment in the study and the mean duration of follow-up (FU) was 10,7±6 years. Interestingly, although no age difference according to sex was observed (males 53±15 years and females 57±15 years, Mann-Whitney p = 0.4), the mean FU was significantly longer in females compared to males (13±7 versus 7±4 years, p = 0.01). A detectable viremia was found at least once during the FU in 11 subjects (27.5%) who had a significantly longer FU compared to always negative ones (16±7 versus 9±6 years p = 0.006). The viral genotype was identified only in four subjects: 2 with genotype 1b, one with genotype 1a and one with genotype 4d. Overall, 3 subjects (7.5%) lost anti-HCV during the FU, none of whom had had a detectable HCV-RNA; although no difference in FU length was observed in subjects who became anti-HCV negative compared to persistently positive ones, those who became negative were significantly younger (40.6 versus 57.4 years; p = 0.04).

All efforts were made to ascertain the potential route of infection for each subject because the amount of inoculum could represent a condition associated with the spontaneous clearance of the infection. The modes of HCV acquisition, not mutually exclusive, are reported in Table 1. It is noteworthy that intravenous drug use (IDU) and occupational exposure (e.g. nursing and medical profession) were uncommon (3 subjects  = 7.5% and 4 subjects  = 10%, respectively), while the majority of subjects reported at least one surgical intervention: 18 subjects (44%) reported a major and 11 (27%) a less invasive surgical procedure (e.g. arthroscopy or laparoscopy). Also a history of blood and/or blood product transfusion or the presence of sexual partner or a family member with known HCV infection were quite common (14 subjects, 34% for each risk factor; Table 1). Subjects with history of surgery were significantly older (59.6 versus 39 years; p = 0.001) while IDU subjects were younger (40 versus 57 years, p = 0.05) compared to all other subjects. No correlation between risk factors and history of detectable viremia or loss of anti-HCV was found.

### IL28B results

**Figure 1:**
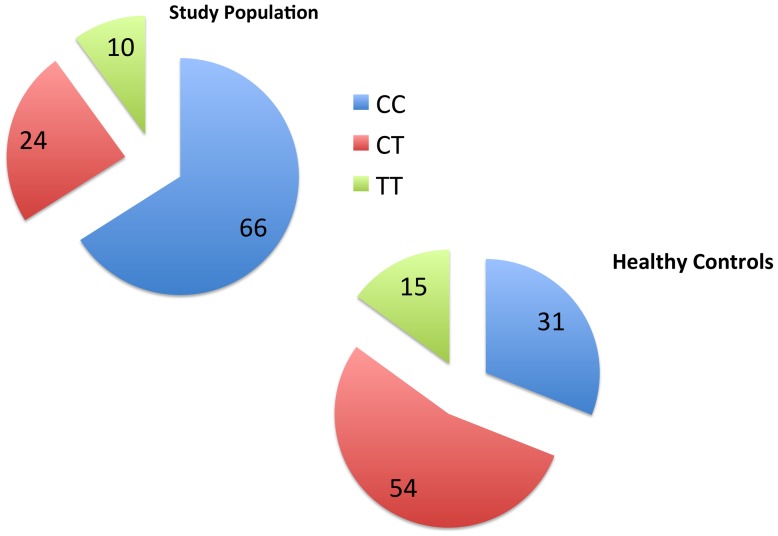
IL28B genotype distribution among 41 subjects who spontaneously cleared HCV infection and 134 healthy controls from the same geographical area (see text). Footnote: Comparison between study subjects and healthy controls: χhi square p<0.001

IL28B rs12979860 genotype was characterized in all subjects. Overall, CC genotype was significantly more frequent in the study population compared to controls (27/41 = 66% among study group subjects versus 42/134 = 31% among controls), while on the opposite, CT/TT genotypes were more frequently found among controls (92/134 = 68% versus 14/41 = 34% ; χ^2^ p<0.0001, figure 1). This finding supports the hypothesis that a segregation of CC genotype occurs in subjects who spontaneously cleared HCV infection. Due to the distribution of the two rs12979860 alleles combination in this population, for further analysis IL28B genotypes were collapsed into a recessive model and comparisons were performed as CC versus non-CC genotypes.

We explored whether in subjects with spontaneous resolution, IL28B polymorphism was associated with different risk factors, but no such association could be demonstrated (data not shown).

The characteristics of the study subjects according to rs1279860 genotype are reported in Tale 2. A significant correlation between sex and IL-28B genotype was observed (Table 2). Interestingly, the majority of the CC subjects were females (21, 78%) while all TT subjects were males (4, 100%; χ^2^ test, p = 0.02). In addition, no correlation of IL28B genotype was found with age, levels of cholesterol or tryglicerides, previous RNA detection or anti-HCV negativization. Only BMI higher than 25 Kg/cm^2^ was significantly more frequent among subjects with CT/TT genotypes (Table 2).

**Table 2:** Characteristics of 41 subjects who spontaneously cleared HCV infection according to rs12979860 genotype. Univariate logistic regression analysis.

	CC Genotype# (27)	CT/TT genotypes# (14)	P
Female sex n. (%)	21 (78%)	6 (43%)	0.02
Age years (mean ± SD)	54.7±16	56,1±15	0.6
BMI Kg/cm^2^ (mean ± SD)	23±3	25±4	0.06
BMI ≥25 Kg/cm^2^ n. (%)	9 (33%)	9 (64%)	0.05
Cholesterol: Total mg/dl (mean ± SD)	195±42	203±51	0.6
HDL mg/dl (mean ± SD)	55±10	50±14	0.25
LDL mg/dl (mean ± SD)	118±36	138±42	0.13
Tryglicerides mg/dl (mean ± SD)	113±48	143±63	0.10
Steatosis n (%)	15 (56%)	11 (79%)	0.14
Previous HCV-RNA positive n. (%)	6 (22%)	5 (36%)	0.35
Anti-HCV negativization during FU n. (%)	3 (11%)	0	0.19

#: IL28B genotypes were collapsed into a recessive model and comparisons were performed as CC versus non-CC genotypes (see text).

Among individuals with CC genotype, 15 subjects (56%) were confirmed by liver biopsy and/or by ultrasound to have evidence of steatosis, compared to 11 out of 14 non-CC subjects (79%; χ^2^ test, p = 0.14), thus no correlation of IL28B genotype with steatosis was observed.

### Assessment of steatosis

All patients underwent ultrasound examination, while liver biopsy was performed in the 6 patients with raised ALT levels. In all examined biopsies, only minimal portal infiltration was observed without evidence of fibrosis. Overall, 25 patients (66%) had evidence of steatosis. Diagnosis of steatosis was based on the ultrasound evidence of at least two out of five main characteristics typically associated with hepatic steatosis (see methods). The most frequently observed characteristics were: liver-kidney ultrasound contrast; parenchymal brightness (95% each) and bright vessel wall (84%). All five criteria were recorded in three patients (16%), four criteria in 7 (37%), three criteria in 8 (45%) and two criteria in only one subject (5%). Overall, diagnosis of steatosis relied on the presence of at least 3 main criteria in 95% of steatosis cases [17].

Among individuals with CC genotype, 15 subjects (56%) had evidence of steatosis, compared to 11 out of 14 non-CC subjects (79%; χ^2^ test, p = 0.14), thus no correlation of IL28B genotype with steatosis was observed. As expected, subjects with steatosis were significantly older compared to those without steatosis (60±15 years versus 50±14 years; p = 0.03), had significantly higher MBI, triglycerides, total and LDL cholesterol levels (Table 3). However, on multivariate analysis, only higher BMI remained a significant predictor of steatosis (Table 4).

**Table 3:** Characteristics of the 41 subjects with spontaneous resolution of HCV infection enrolled in the study according to steatosis. Univariate logistic regression analysis.

	Steatosis		P value
	Yes	No	
Age years (mean ± SD)	60±15	50±14	0.03
BMI Kg/m^2^	26±3	21±3	0.004
BMI ≥25 Kg/m^2^	16	2	0.003
Cholesterol mg/dl			
Total	208±42	181±46	0.03
HDL	55±11	50±13	0.15
LDL	138±35	102±36	0.006
Triglycerides mg/dl	141±53	94±46	0.007
Glycemia mg/dl	96±16	91±10	0.09
IL28B rs 12979860 CC versus TC-TT #	15/11	12/3	0.14

#: IL28B genotypes were collapsed into a recessive model and comparisons were performed as CC versus non-CC genotypes (see text).

**Table 4:** Multivariate analysis of factors associated to liver steatosis in the 41 subjects who spontaneously cleared HCV infection.

	Odds Ratio	95% CI	P value
MBI ≤25 Kg/m^2^	0.13	0.02–0.8	0.03
LDL-Cholesterol ≤160 mg/dl	0.3	0.4–13	0.3
Triglycerides ≤170 mg/dl	0.4	0.1–37	0.1
Age ≤50	1.8	0.3–10	0.5

Interestingly, although subjects with steatosis (table 3) and male subjects had a significantly higher BMI (BMI of males 26±3 versus females 23±4 Kg/m^2^, p = 0.01) no correlation between gender and steatosis was observed (steatosis in males 71% versus females 59%; χ^2^ p = 0.4).

## Discussion

Genome Wide Association Studies (GWAS) have described single nucleotides polymorphisms (SNPs) on chromosome 19 that were highly predictive of spontaneous resolution of acute hepatitis C infection [19]. Our results, exploring the rs12979860 SNP in the region of the IL28B, indicate an increased prevalence of IL28B CC genotype among subjects who spontaneously cleared HCV infection compared to healthy control subjects (χ^2^ <0.001). To our knowledge, this is the first study reporting the association between rs12979860 CC genotype and spontaneous clearance of HCV infection in an unselected Italian population. Our data support the hypotheses that: 1) a segregation of CC genotype occurs in subjects who spontaneously cleared HCV infection, 2) CC genotype could play a protective role against the development of chronic HCV infection, and 3) IL28B SNP may have a primary importance in establishing the prognosis of patients affected by acute hepatitis C [15], [19].

In Caucasian and Afro-American cohorts of HCV patients, rs12979860 CC genotype was associated with spontaneous virus clearance [14], [19], while in Australian and in Chinese patients a strong [9] and a very strong [20] association with rs8099917 TT genotype were reported, respectively. However, a linkage disequilibrium between rs8099917 and rs12979860 exists [9], and in has been shown that inclusion of many SNPs in the analysis did not improve the prediction of spontaneous HCV clearance in Caucasian patients [20], thus rs12979860 may represent a suitable means of evaluating the association of IL28B gene variants with spontaneous clearance of HCV-RNA.

HCV clearance versus persistence is almost always determined during the first months following infection, although cases of late spontaneous clearance have been documented [21]. Based on their medical records and laboratory reports, we know that 29% of the enrolled subjects had a detectable HCV viremia 7–12 years before entry in the study. Unfortunately, we do not know the exact timing of their HCV infection, therefore we cannot speculate on the contribution of IL28 genotype to the rapidity of HCV clearance. Interestingly, we found that 3 (7.5%) female subjects carrying CC genotype lost anti-HCV during the FU, and they were significantly younger compared to persistently anti-HCV positive ones (40.6 years versus 57.4 years; p = 0.04). This result confirms that eradication of HCV may occur [22], and suggests that this biological event is more probable if the subjects are young and have spontaneously cleared serum HCV-RNA.

It has been previously suggested that type and size of the inoculum may affect the outcome of HCV infection [23]. The majority of the examined subjects (27 out of 41, 66%) acquired the infection through a small/very small inoculum (i.e. parenteral unapparent route) while only 34% were possibly exposed to a large inoculum via blood transfusion. We did not find any correlation between mode of acquisition and IL28B genotypes, but we cannot exclude that the number of recruited subjects could be too small for definitive conclusions to be drawn.

On a demographic ground, female gender was prevalent in our study population (64%). In addition, females were significantly more frequently CC compared to males (75% versus 22%, Chi square test p = 0.02). The association between female sex and self-limiting acute hepatitis C has already been reported [8], [24], thought the reasons for this association remain largely unknown. It has been recently suggested a role for toll like receptor 7, that is involved in recognition of viral products (single stranded RNA) and activation of innate immunity. Stimulation of toll like receptor 7 determines, via interferon stimulating genes (ISG), a significantly higher IFN-alpha responses in females compared to males [25]. In addition, in carriers of the rs12979869 C allele, serum levels of IFN-λ-2/3 were significantly higher compared to carriers of the TT genotype, which may predispose to spontaneous resolution of HCV infection [26]. Thus, the spontaneous resolution of HCV infection that occurred in our population is probably consequent to being prevalently female and having the IL28B CC genotype. Interestingly, the mean duration of the follow-up was significantly longer in female subjects compared to males (p = 0.01), although the mean age was similar in both genders. This finding simply underlines a well-recognized sex difference in the utilization of health care services [27].

A different explanation of the interplay between IL28B and HCV clearance may rely on the interaction between the virus and lipids, particularly apo-lipoproteins B (apoB) and E (apoE). Such interaction occurs at multiple stages of virus lifecycle, including replication on lipid droplets, assembly, release and viral entry and, most importantly, infectious lipoviral particles (LVP) formation [28]. This last step is of particular importance since it seems that the presence of increased apoE on the surface of LVP is associated with increased infectivity, possibly by facilitating interaction with lipoprotein receptors on hepatocytes [29]. There is robust evidence of the association of CC genotype with lower levels of apoE but higher levels of apoB and LDL [30]. Thus, in CC patients a less infectious virus may be produced during replication, and this may explain why they are more likely to clear infection, given that virus infectiousness is likely much more important at the time of acute exposure, when viral spread is rapidly progressing, than during interferon treatment [31].

Finally, HCV displays some important interactions with lipid metabolism; in particular impaired lipoprotein secretion and decreased fatty acid oxidation seem to be critical events leading to fatty liver induced by HCV and the core protein of the virus is capable and sufficient to produce such effects [32]–[33]. Therefore, it is not surprising that steatosis is observed in nearly 50% of HCV patients, which is up to twice as may compared to hepatitis B.

In addition and more recently, rs12979860 polymorphism of IL28B has been associated with the presence and severity of steatosis in Caucasian patients, particularly those infected with genotype-1 chronic infection. In particular, patients with T allele seem to have significantly more steatosis compared to CC patients, suggesting potential genetic risk pathways to this metabolic disturbance in HCV patients and that both host and viral factors concur to its development [33]–[34].

Interestingly, we did not find any association between the presence of T allele and steatosis, as demonstrated either by US or biopsy (Table 3), while on multivariate analysis we found a strong correlation between steatosis and BMI (Table 4). Our finding is of particular interest because in the present population the virus was not actively replicating, which led us to hypothesize that the role IL28B polymorphism plays in regulating the development of hepatic steatosis in HCV patients is not independent from, but presumably mediated by the poor control on HCV replication that patients with the unfavourable IL28B genotype exert and requires the presence of actively replicating HCV virus to be played. On the opposite, in subjects without actively replicating HCV infection, the mechanism(s) that regulate the occurrence of steatosis seem to be independent from IL28B polymorphism but purely related to the lipid disturbance occurring in non-alcoholic fatty liver disease, which remains one of the most important clinical features of metabolic syndrome [35].

Unfortunately we were not able to evaluate the HCV genotype effect on liver steatosis since we were able to genotype only four patients, and this could represent a limitation of our study. On one side, recent findings suggest that viral factors are relevant in the induction of fatty changes in HCV-infected persons, especially those with genotype 3 infection, in whom steatosis is more common and more severe and the severity of lipid accumulation in hepatocytes correlates with the level of HCV replication [32], [36]–[37]. On the other side, the association between steatosis and IL28B is reported mainly among patients with genotype 1 infection. However, in genotype 3 patients reversal of steatosis is commonly observed after successful eradication of HCV infection [37] therefore we can speculate that in our patients, who had cleared HCV infection on average 10.7 years before enrolment in the study, the contribution of HCV to the actual presence of steatosis was probably negligible even if they were infected by genotype 3. On the other hand, a significant association of IL28B with fatty changes of the liver irrespective of HCV genotype was recently reported in japanese patients with active HCV infection (11). Finally, and most importantly, the spontaneous clearance of HCV, as was observed in our patients, usually occurs within the first 6–24 weeks from infection [38]–[39], thus we can speculate that in such a short span of time the influence each HCV genotype may lead to the occurrence of steatosis was probably minimal. Therefore, the unavailability of HCV genotype probably does not affect the relevance of our finding of the lack of association between steatosis and IL28B in the absence of viral replication.

In conclusion, our results indicate that female subjects carrying the CC genotype are significantly more represented among HCV infected Italian patients who spontaneously cleared the infection. In addition, among these subjects, the presence of significant liver steatosis did not correlate with the IL28B genotype but was solely related to the occurrence of high BMI, which is a typical feature of the metabolic syndrome. This finding suggests that IL28B polymorphism may play some role in favouring the development of hepatic steatosis in CT/TT patients only in the presence of active HCV replication, and such lipid metabolism disturbance is presumably mediated by the poor control on HCV replication that patients with the unfavourable IL28B genotype exert. On the opposite, in subjects who have cleared the infection, the mechanism(s) inducing liver steatosis seem to be independent from IL28B profile.

## References

[1] Perz JF, Farrington LA, Pecoraro C, Hutin YJ, Armstrong GL (2004) Estimated global prevalence of hepatitis C virus infection, Boston, MA, USA.

[2] Kim AY, Kuntzen T, Timm J, Nolan BE, Baca MA, et al (2011) Spontaneous control of HCV is associated with expression of HLA-B 57 and preservation of targeted epitopes. Gastroenterology 140: 686–696. 20875418 10.1053/j.gastro.2010.09.042 PMC3021586

[3] Barrett S, Goh J, Coughlan B, Ryan E, Stewart S, et al (2001) The natural course of hepatitis C virus infection after 22 years in a unique homogenous cohort: spontaneous viral clearance and chronic HCV infection Gut. 49: 423–430. 10.1136/gut.49.3.423 PMC1728443 11511566

[4] Diepolder HM, Zachoval R, Hoffmann RM, Wierenga EA, Santantonio T, et al (1995) Possible mechanism involving T–lymphocyte response to non-structural protein 3 in viral clearance in acute hepatitis C virus infection. Lancet 346: 1006–1007. 7475549 10.1016/s0140-6736(95)91691-1

[5] Thio CL (2008) Host genetic factors and antiviral immune responses to hepatitis C virus Clin. Liver Dis 12: 713–726. 10.1016/j.cld.2008.03.002 PMC2597299 18625436

[6] Wietzke-Braun P, Manhardt LB, Rosenberger A, Uy A, Ramadori G, et al (2007) Spontaneous elimination of hepatitis C virus infection: a retrospective study on demographic, clinical, and serological correlates. World J Gastroenterol 13: 4224–9. 17696252 10.3748/wjg.v13.i31.4224 PMC4250622

[7] Thomas DL, Astemborski J, Rai RM, Anania FA, Schaeffer M, et al (2000) The natural history of hepatitis C virus infection: host, viral, and environmental factors JAMA. 284: 450–6. 10.1001/jama.284.4.450 10904508

[8] Micallef JM, Kaldor JM, Dore GJ (2006) Spontaneous viral clearance following acute hepatitis C infection: A systematic review of longitudinal studies J Viral Hepat. 13: 34–41. 10.1111/j.1365-2893.2005.00651.x 16364080

[9] Rauch A, Kutalik Z, Descombes P, Cai T, Di Iulio J, et al (2010) Genetic variation in IL28B is associated with chronic hepatitis C and treatment failure: a genome-wide association study Gastroenterology. 138: 1338–1345. 10.1053/j.gastro.2009.12.056 20060832

[10] Clark PJ, Thompson AJ, Zhu Q, Vock DM, Zhu M, et al (2012) The association of genetic variants with hepatic steatosis in patients with genotype 1 chronic hepatitis C infection Dig Dis Sci. 57: 2213–21. 10.1007/s10620-012-2171-y PMC3518927 22543885

[11] Ohnishi M, Tsuge M, Kohno T, Zhang Y, Abe H, et al (2012) IL28B polymorphism is associated with fatty change in the liver of chronic hepatitis C patients J Gastroenterol. 47: 834–44. 10.1007/s00535-012-0550-y 22350701

[12] Urban TJ, Thompson AJ, Bradrick SS, Fellay J, Schuppan D, et al (2010) IL28B genotype is associated with differential expression of intrahepatic interferon-stimulated genes in patients with chronic hepatitis C. Hepatology. 52: 1888–96. 10.1002/hep.23912 PMC3653303 20931559

[13] Ochi H, Maekawa T, Abe H, Hayashida Y, Nakano R, et al (2011) IL-28B predicts response to chronic hepatitis C therapy–fine-mapping and replication study in Asian populations. J Gen Virol 92: 1071–81. 21228123 10.1099/vir.0.029124-0

[14] Tillmann HL, Thompson AJ, Patel K, Wiese M, Tenckhoff H, et al (2010) A polymorphism near IL28B is associated with spontaneous clearance of acute hepatitis C virus and jaundice Gastroenterology. 139: 1586–92. 10.1053/j.gastro.2010.07.005 20637200

[15] Di Iulio J, Ciuffi A, Fitzmaurice K, Kelleher D, Rotger M, et al (2011) Estimating the net contribution of interleukin-28B variation to spontaneous hepatitis C virus clearance Hepatology. 53: 1446–54. 10.1002/hep.24263 PMC3128709 21360716

[16] Thompson AJ, Muir AJ, Sulkowski MS, Ge D, Fellay J, et al (2010) Interleukin-28B polymorphism improves viral kinetics and is the strongest pretreatment predictor of sustained virologic response in genotype 1 hepatitis C virus Gastroenterology. 139: 120–129. 10.1053/j.gastro.2010.04.013 20399780

[17] Hamaguchi M, Kojima T, Itoh Y, Harano Y, Fujii K, et al (2007) The severity of ultrasonographic findings in nonalcoholic fatty liver disease reflects the metabolic syndrome and visceral fat accumulation. Am J Gastroenterol 102: 2708–15. 17894848 10.1111/j.1572-0241.2007.01526.x

[18] Fakhrai-Rad H, Pourmand N, Ronaghi M (2002) Pyrosequencing: an accurate detection platform for single nucleotide polymorphisms Hum Mutat. 19: 479–85. 10.1002/humu.10078 11968080

[19] Thomas DL, Thio CL, Martin MP, Qi Y, Ge D, et al (2009) Genetic variation in IL28B and spontaneous clearance of hepatitis C virus Nature. 461: 798–801. 10.1038/nature08463 PMC3172006 19759533

[20] Rao HY, Sun DG, Jiang D, Yang RF, Guo F, et al (2012) IL28B genetic variants and gender are associated with spontaneous clearance of hepatitis C virus infection J Viral. Hepat19: 173–81. 10.1111/j.1365-2893.2011.01497.x 22329371

[21] Cox AL, Netski DM, Mosbruger T, Sherman SG, Strathdee S, et al (2005) Prospective evaluation of community-acquired acute-phase hepatitis C virus infection. Clin Infect Dis 40: 951–958. 15824985 10.1086/428578

[22] Fujiwara K, Allison RD, Wang RY, Bare P, Matsuura K, et al (2013) Investigation of residual hepatitis C virus in presumed recovered subjects Hepatology. 57: 483–91. 10.1002/hep.25921 PMC4523271 22729600

[23] Alberti A, Chemello L, Benvegnù L (1999) Natural history of hepatitis C. J Hepatol. 31 Suppl 117–24. 10.1016/s0168-8278(99)80369-9 10622555

[24] Van den Berg CH, Grady BP, Schinkel J, van de Laar T, Molenkamp R, et al (2011) Female sex and IL28B, a synergism for spontaneous viral clearance in hepatitis C virus (HCV) seroconverters from a community-based cohort PLoS One. 6: e27555. 10.1371/journal.pone.0027555 PMC3216978 22110669

[25] Berghöfer B, Frommer T, Haley G, Fink L, Bein G, et al (2006) TLR7 ligands induce higher IFN-alpha production in females J Immunol. 177: 2088–2096. 10.4049/jimmunol.177.4.2088 16887967

[26] Langhans B, Kupfer B, Braunschweiger I, Arndt S, Schulte W, et al (2011) Interferon-lambda serum levels in hepatitis C J Hepatol. 54: 859–65. 10.1016/j.jhep.2010.08.020 21145813

[27] Mustard CA, Kaufert P, Kozyrsky A, Mayer T (1998) Sex differences in the use of health care services N Engl J Med. 338: 1678–83. 10.1056/NEJM199806043382307 9614260

[28] Benga WJ, Krieger SE, Dimitrova M, Zeisel MB, Parnot M, et al (2010) Apolipoprotein E interacts with hepatitis C virus nonstructural protein 5A and determines assembly of infectious particles Hepatology. 51: 43–53. 10.1002/hep.23278 20014138

[29] Owen DM, Huang H, Ye J, Gale Jr M (2009) Apolipoprotein E on hepatitis C virion facilitates infection through interaction with low-density lipoprotein receptor Virology. 394: 99–108. 10.1016/j.virol.2009.08.037 PMC2767442 19751943

[30] Li JH, Lao XQ, Tillmann HL, Rowell J, Patel J, et al (2010) Interferon-lambda Genotype and Low Serum Low-Density Lipoprotein Cholesterol Levels in Patients with Chronic Hepatitis C Infection Hepatology. 51: 1904–1911. 10.1002/hep.23592 PMC2921623 20235331

[31] Feld JJ (2012) Interferon responses and spontaneous HCV clearance: Is it all a matter of fat? J Hepatol 57: 3–5. 22521343 10.1016/j.jhep.2012.04.003

[32] Negro F, Sanyal AJ (2009) Hepatitis C virus, steatosis and lipid abnormalities: clinical and pathogenic data Liver Int. 29 (Suppl 2)26–37. 10.1111/j.1478-3231.2008.01950.x 19187070

[33] Bugianesi E, Salamone F, Negro F (2012) The interaction of metabolic factors with HCV infection: does it matter? J Hepatol 56 Suppl 1S56–65. 22300466 10.1016/S0168-8278(12)60007-5

[34] Clark PJ, Thompson AJ, Zhu Q, Vock DM, Zhu M, et al (2012) The Association of Genetic Variants with Hepatic Steatosis in Patients with Genotype 1 Chronic Hepatitis C Infection Dig Dis Sci. 57: 2213–21. 10.1007/s10620-012-2171-y PMC3518927 22543885

[35] McCullough AJ (2011) Epidemiology of the metabolic syndrome in the USA J Dig Dis. 12: 333–340. 10.1111/j.1751-2980.2010.00469.x 21091931

[36] Rubbia-Brandt L, Quadri R, Abid K, Giostra E, Male PJ, et al (2000) Hepatocyte steatosis is a cytopathic effect of hepatitis C virus genotype 3 J Hepatol. 33: 106–115. 10.1016/s0168-8278(00)80166-x 10905593

[37] Kumar D, Farrell GC, Fung C, George J (2002) Hepatitis C virus genotype 3 is cytopathic to hepatocytes. Genotype-specific reversal of hepatic steatosis after sustained response to antiviral therapy Hepatology 36: 1266–72. 10.1053/jhep.2002.36370 12395339

[38] Gerlach JT, Diepolder HM, Zachoval R, Gruener NH, Jung MC, et al (2003) Acute hepatitis C: high rate of both spontaneous and treatment-induced viral clearance Gastroenterology. 125: 80–8. 10.1016/s0016-5085(03)00668-1 12851873

[39] Santantonio T, Medda E, Ferrari C, Fabris P, Cariti G, et al (2006) Risk factors and outcome among a large patient cohort with community-acquired acute hepatitis C in Italy Clin Infect Dis. 43: 1154–9. 10.1086/507640 17029134

